# Supplementary material for: ATP released by intestinal bacteria limits the generation of protective IgA against enteropathogens
Source: Nat Commun. 2019 Jan 16;10:250. doi: 10.1038/s41467-018-08156-z (PMC6335424; doi:10.1038/s41467-018-08156-z)
Supplement: Supplementary file 1 — Supplementary Information [file 41467_2018_8156_MOESM1_ESM.pdf]

Supplementary Information

"ATP released by intestinal bacteria limits the generation of protective IgA against enteropathogens"

Proietti, Perruzza et al.

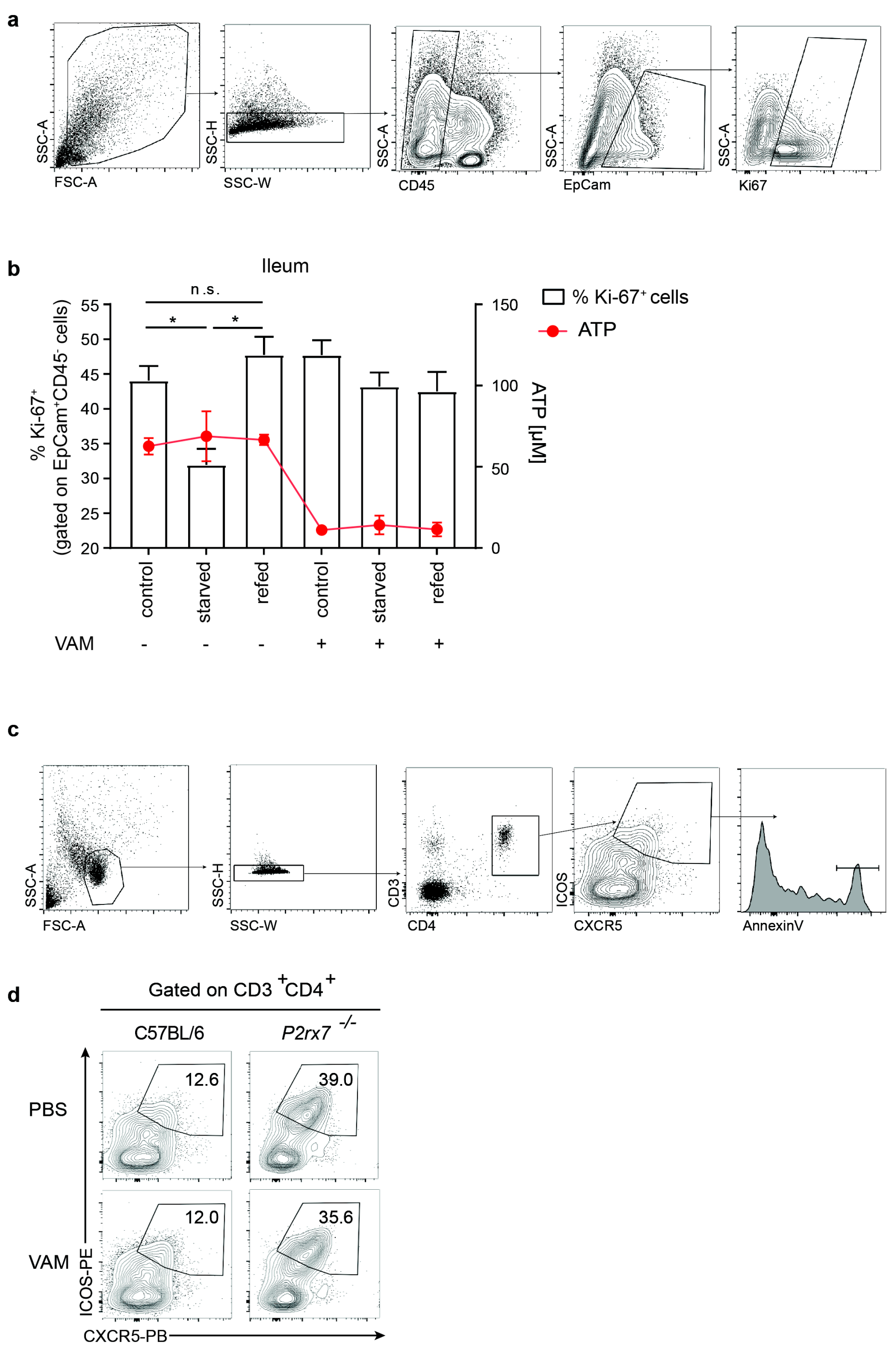

Supplementary Figure 1

Epithelial turnover induced by starvation-refeeding in SPF mice does not affect eATP levels. Staining of Tfh cells in flow cytometry.

(**a**) Gating strategy used to analyse Ki-67$^+$ ileal epithelial cells (EpCam$^+$CD45$^-$) shown in panel b. (**b**) Ileal epithelial cells were purified from SPF mice either untreated or treated for 15 days with VAM. Mice were fed ad libitum (control), starved for 36 h or starved for 36 h and re-fed with normal diet for 24 h. Bars show percentage of Ki-67$^+$ cells and red line the corresponding concentration of ileal eATP. Two-tailed Mann–Whitney U-tests. *p < 0.05; n.s., non-significant. One representative experiment out of two is shown. (**c**) Gating strategy used to analyse Annexin V$^+$ Tfh cells (CD3$^+$CD4$^+$CXCR5$^+$ICOS$^+$) from PPs shown in Fig. 1g. The same strategy was used to stain Tfh cells in Fig. 3a, 4b and panel d in this figure. (**d**) Representative dot plots of Tfh cells from PPs of WT (C57BL/6) and *P2rx7*$^{-/-}$ mice at 3 h after gavage with PBS or VAM.

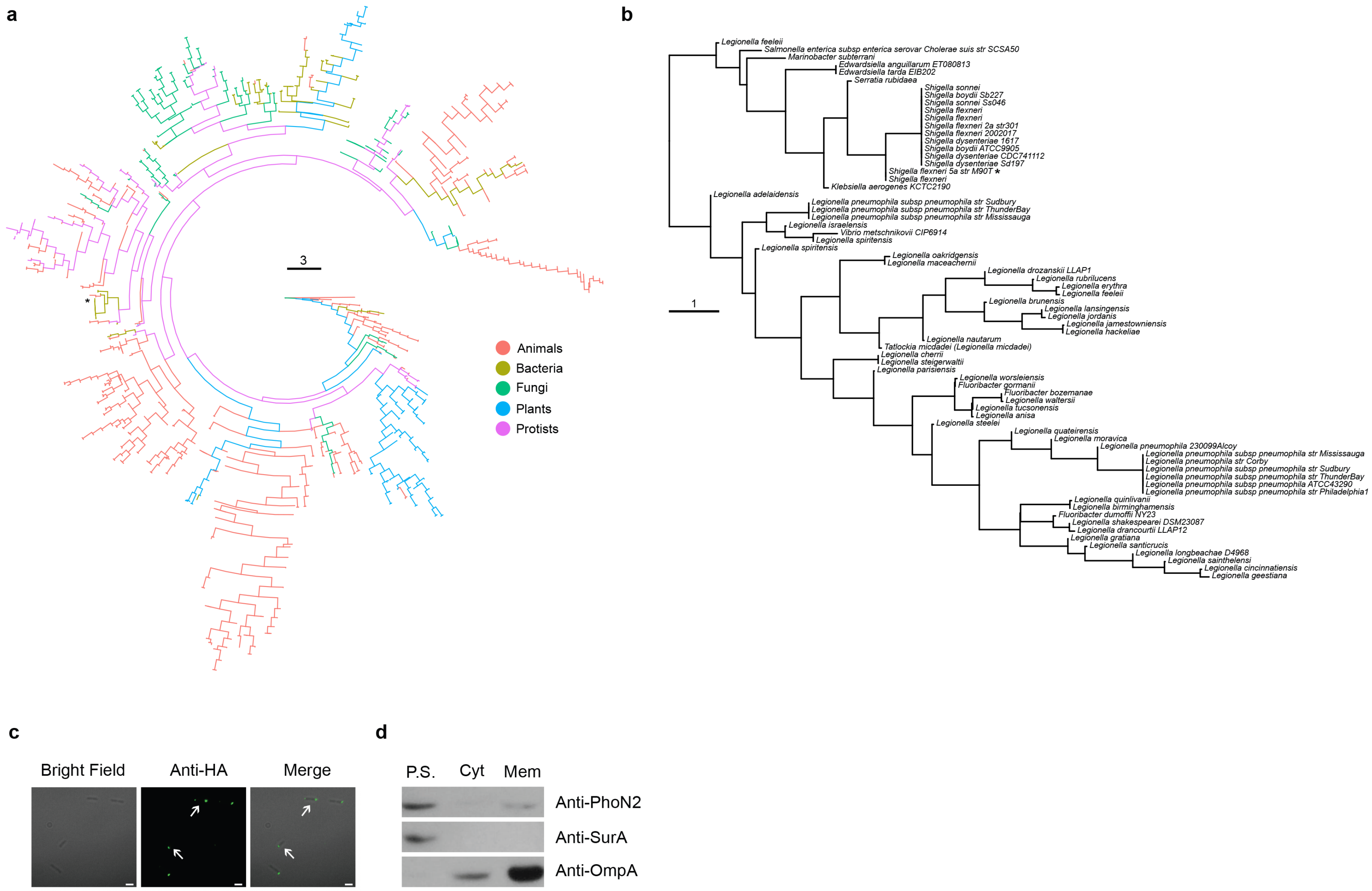
a
3
Animals
Bacteria
Fungi
Plants
Protists
*
b
1
Legionella feeleii
Salmonella enterica subsp enterica serovar Cholerae suis str SCSA50
Marinobacter subterrani
Edwardsiella anguillarum ET080813
Edwardsiella tarda EIB202
Serratia rubidaea
Shigella sonnei
Shigella boydii Sb227
Shigella sonnei Ss046
Shigella flexneri
Shigella flexneri
Shigella flexneri 2a str301
Shigella flexneri 2002017
Shigella dysenteriae 1617
Shigella boydii ATCC9905
Shigella dysenteriae CDC741112
Shigella dysenteriae Sd197
Shigella flexneri 5a str M90T *
Shigella flexneri
Klebsiella aerogenes KCTC2190
Legionella adelaidensis
Legionella pneumophila subsp pneumophila str Sudbury
Legionella pneumophila subsp pneumophila str ThunderBay
Legionella pneumophila subsp pneumophila str Mississauga
Legionella israelensis
Vibrio metschnikovii CIP6914
Legionella spiritensis
Legionella spiritensis
Legionella oakridgensis
Legionella maceachernii
Legionella drozanskii LLAP1
Legionella rubrilucens
Legionella erythra
Legionella feeleii
Legionella brunensis
Legionella lansingensis
Legionella jordanis
Legionella jamestowniensis
Legionella hackeliae
Legionella nautarum
Tatlockia micdadei (Legionella micdadei)
Legionella cherrii
Legionella steigerwaltii
Legionella parisiensis
Legionella worsleiensis
Fluoribacter gormanii
Fluoribacter bozemanae
Legionella waltersii
Legionella tucsonensis
Legionella anisa
Legionella steelei
Legionella quateirensis
Legionella moravica
Legionella pneumophila 230099Alcoy
Legionella pneumophila subsp pneumophila str Mississauga
Legionella pneumophila str Corby
Legionella pneumophila subsp pneumophila str Sudbury
Legionella pneumophila subsp pneumophila str ThunderBay
Legionella pneumophila subsp pneumophila ATCC43290
Legionella pneumophila subsp pneumophila str Philadelphia1
Legionella quinlivanii
Legionella birminghamensis
Fluoribacter dumoffii NY23
Legionella shakespearei DSM23087
Legionella drancourtii LLAP12
Legionella gratiana
Legionella santicrucis
Legionella longbeachae D4968
Legionella sainthelensi
Legionella cincinnatiensis
Legionella geestiana
c
Bright Field
Anti-HA
Merge
d
P.S.
Cyt
Mem
Anti-PhoN2
Anti-SurA
Anti-OmpA

Supplementary Figure 2

Phylogenetic tree of ectonucleotidases and analysis of apyrase expression in *E. coli*

(**a**) Phylogenetic tree of apyrases and ectonucleoside triphosphate diphosphohydrolases in all domains of life and (**b**) among bacteria. (**c**) *PhoN2*::HA fusion*,* encoding *Shigella flexneri's* periplasmic ATP-diphosphohydrolase, was cloned into plasmid pHND10 (a derivative of pBAD28) under the control of a L-arabinose inducible promoter. Bright field image of pHND10 carrying DH10b *E. coli* and polar localization of PhoN2 by staining with FITC-labeled anti-HA mab (arrows) and merged image. Bacteria were grown in the presence of L-arabinose to induce *phoN2*::HA expression. Scale bar: 2 µm. (**d**) Periplasmic localization of PhoN2 expressed by DH10b *E. coli* by Western blot analysis of periplasmic space (P.S.), cytosol (Cyt) and membrane (Mem) fractions. Blots were revealed by anti-PhoN2, -SurA and -OmpA antibodies, as indicated.

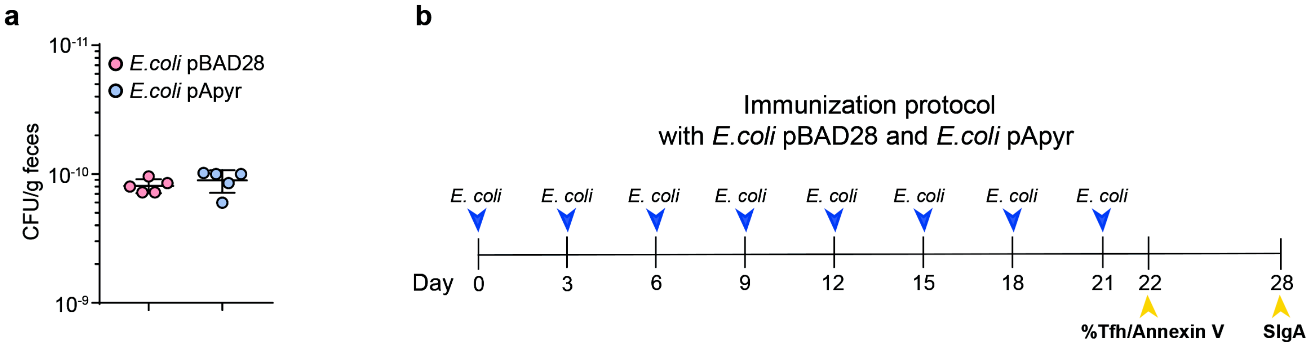

Supplementary Figure 3

Fecal bacterial loads of *E.coli*$^{\text{pBAD28}}$ and *E.coli*^{pApyr} and immunization protocol.

(**a**) Fecal bacterial loads (CFU) in SPF mice at 12 h after gavaging with 10^{10} CFUs of *E. coli*^{pBAD28} and *E. coli*^{pApyr}. Fecal samples were plated in LB agar supplemented with 30 μg/ml chloramphenicol and 100 μg/ml ampicillin in order to select plasmid-harboring *E. coli*. One representative experiment out of three is shown. (**b**) Diagram showing the immunization protocol with *E.coli*$^{\text{pBAD28}}$ and *E.coli*^{pApyr}.

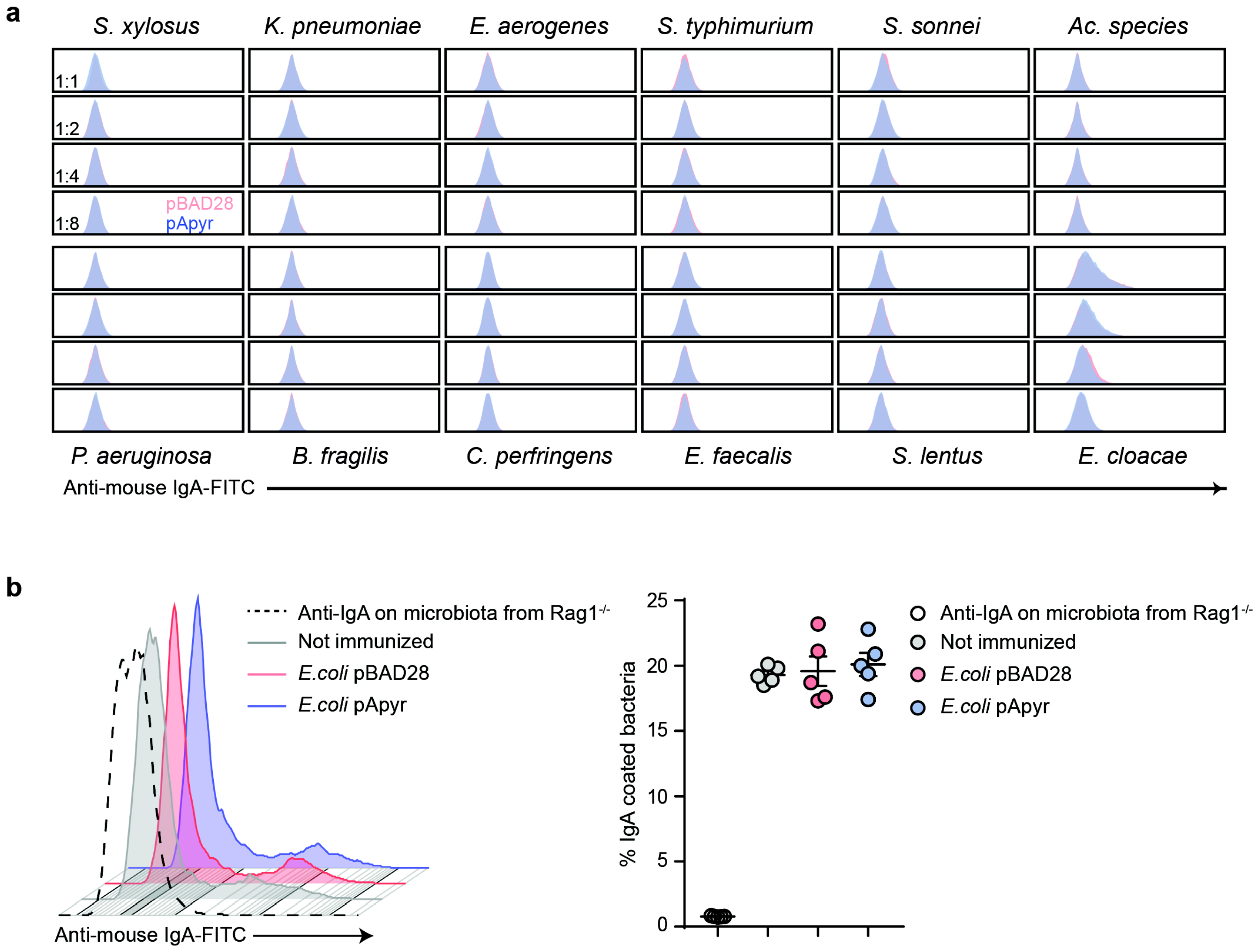

Supplementary Figure 4
Lack of cross-reactivity of SIgA induced by *E. coli* immunization
(**a**) Overlays of flow cytometry profiles on the indicated bacterial species of intestinal wash dilutions from mice immunized with *E. coli*^{pBAD28} (red histograms) or *E. coli*^{pApyr} (light blue histograms). (**b**) Overlay of flow cytometry profiles (left panel) and relative statistical analysis of IgA coated microbiota by intestinal washes of mice immunized with *E. coli*^{pBAD28} or *E. coli*^{pApyr}. *Rag1*$^{-/-}$ microbiota was used as negative control for the secondary antibody. One representative experiment out of two is shown.

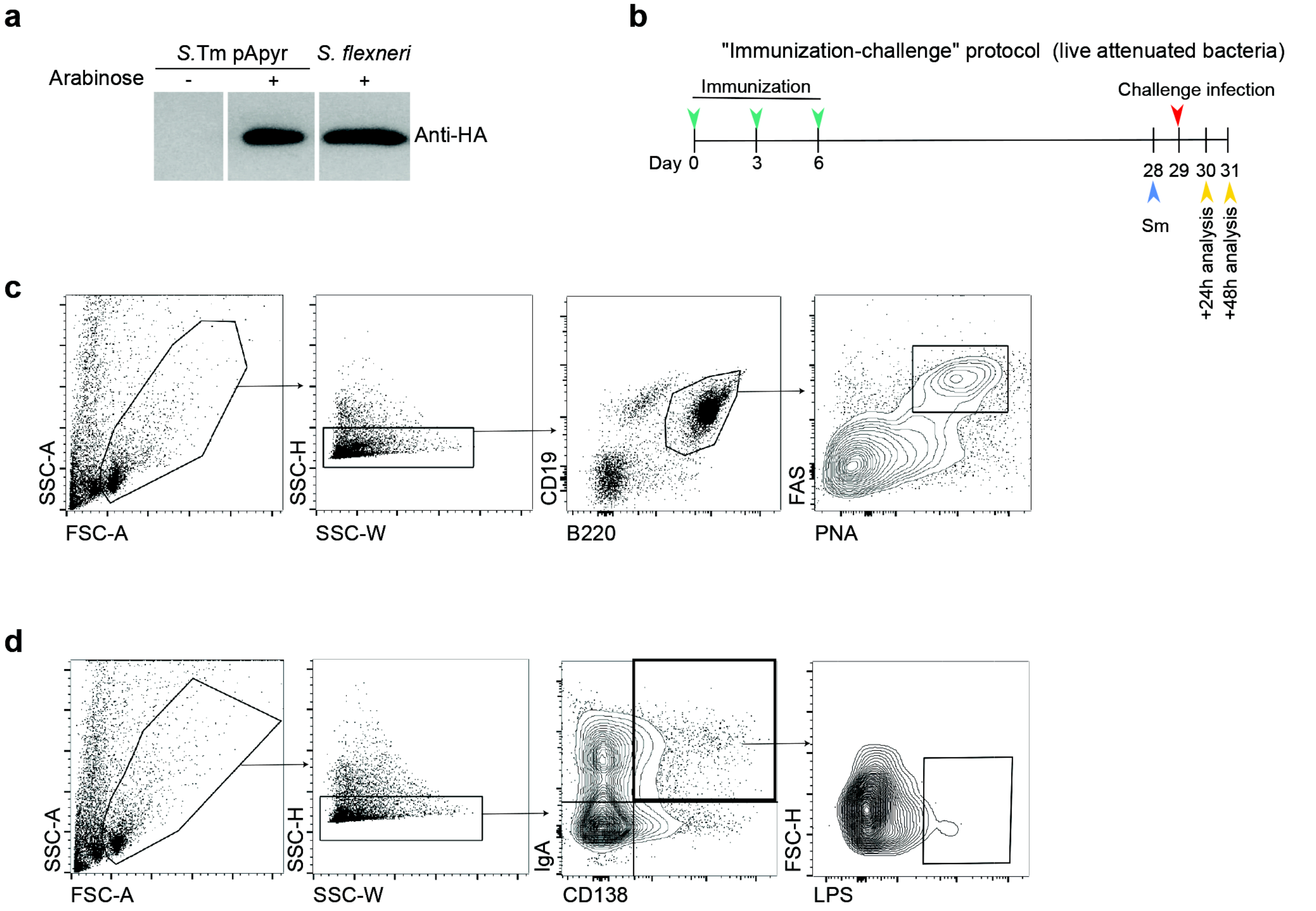

Supplementary Figure 5

Analysis of Phon2 expression by *S*.Tm^{pApyr} and diagram of immunization-challenge protocol. Staining of GC B and plasma cells in flow cytometry.

(a) Western blot showing Phon2 expression by *S*.Tm^{pApyr} grown in the absence or presence of L-arabinose. As control blot, we used an extract of *S. flexneri* mutant strain HNDHA10 carrying the phoN2::HA fusion protein. (**b**) Diagram showing the immunization-challenge protocol with live-attenuated bacteria. At day 0, 3 and 6 mice were gavaged with the different transformants. On day 28, Sm was administered (25 mg i.g.); 24 h later (day 29) mice were infected with 10^8 CFU of *S*.Tm^{WT}. At 24 or 48 h post infection, mice were euthanized for analysis. (**c**) Gating strategy used to analyse GC B cells ($CD19^+B220^+PNA^+FAS^+$) shown in Fig. 4b. (**d**) Gating strategy used to analyse plasma cells specific for S.Tm LPS ($IgA^+CD138^+LPS^+$) shown in Fig. 4c.

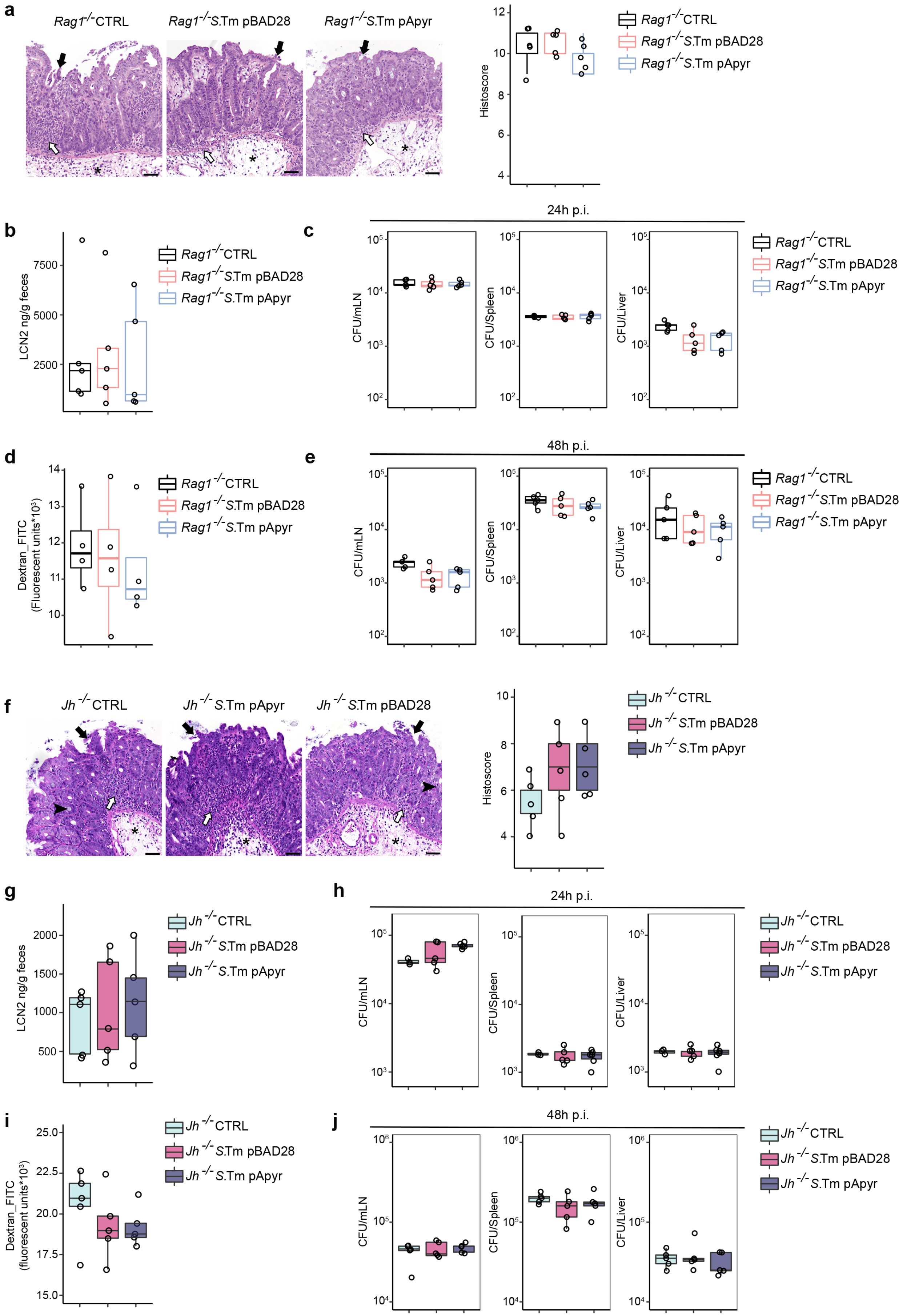
a
Rag1-/-CTRL
Rag1-/-S.Tm pBAD28
Rag1-/-S.Tm pApyr
Histoscore
b
LCN2 ng/g feces
c
24h p.i.
CFU/mLN
CFU/Spleen
CFU/Liver
d
Dextran_FITC (Fluorescent units*10³)
e
48h p.i.
f
Jh-/-CTRL
Jh-/-S.Tm pApyr
Jh-/-S.Tm pBAD28
g
h
i
Dextran_FITC (fluorescent units*10³)
j

Supplementary Figure 6
Lack of apyrase effect in *Rag1*$^{-/-}$ and *Jh*$^{-/-}$ mice
Rag1$^{-/-}$ and *Jh*$^{-/-}$ mice were either gavaged with PBS (CTRL) or vaccinated with *S*.Tm^{pBAD28}or *S*.Tm^{pApyr}. After pre-treatment with streptomycin, mice were infected with 10^8 CFU of *S*.Tm^{WT} and analysed 24 h and 48 h later. (**a, f**) Representative H&E sections of the cecum at 24 h post-infection and statistical analysis of histopathological scores in the indicated mice. Star: submucosal edema; white arrow: neutrophils aggregates; black arrow: epithelial defects; arrowhead: goblet cells. Scale bar: 50 μm. (**b, g**) Fecal LCN2 quantification 24 h post infection with *S*.Tm^{WT}. (**c, h**) Pathogen loads (CFU) in mLN, spleen and liver 24 h after infection. (**d, i**) Intestinal permeability to FITC-dextran 24 h after infection with *S*.Tm^{WT}. Serum levels of 70-kDa FITC-dextran were assessed 4 h after gavage. (**e, j**) Pathogen loads (CFU) in mLN, spleen and liver 48 h after infection. One representative experiment out of three is shown.

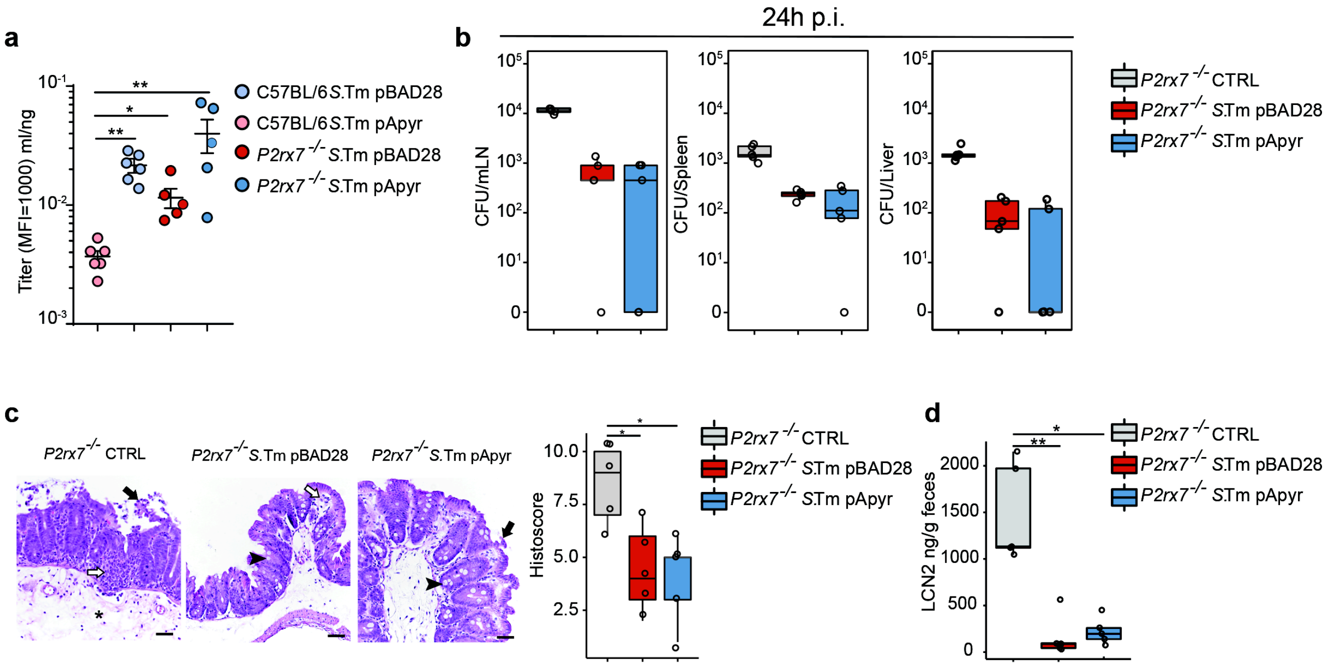

Supplementary Figure 7

Undistinguishable SIgA response and susceptibility to *S.*Tm^{WT} infection in *P2rx7*$^{-/-}$ mice upon immunization with *S.*Tm transformants

Wild-type and *P2rx7*$^{-/-}$ mice were either gavaged with PBS (CTRL) or vaccinated with *S.*Tm^{pBAD28}or *S.*Tm^{pApyr}. After pre-treatment with Sm, mice were infected with 10^8 CFU of *S.*Tm^{WT} and analysed 24 h later. (**a**) Intestinal lavage IgA titer in WT littermates (C57BL/6) and *P2rx7*$^{-/-}$ mice immunized with *S.*Tm^{pBAD28} or *S.*Tm^{pApyr}. (**b**) Pathogen loads (CFU) in mLN, liver and spleen 24 h after infection. (**c**) Representative H&E sections of the cecum at 24 h post-infection and statistical analysis of histopathological scores. Star: submucosal edema; white arrow: neutrophils aggregates; black arrow: epithelial defects; arrowhead: goblet cells. Scale bar: 50 µm. (**d**) Fecal LCN2 quantification 24 h post infection with *S.*Tm^{WT}. Kruskal–Wallis with Dunn's post-test. *p < 0.05, **p < 0.01. One representative experiment out of three is shown.

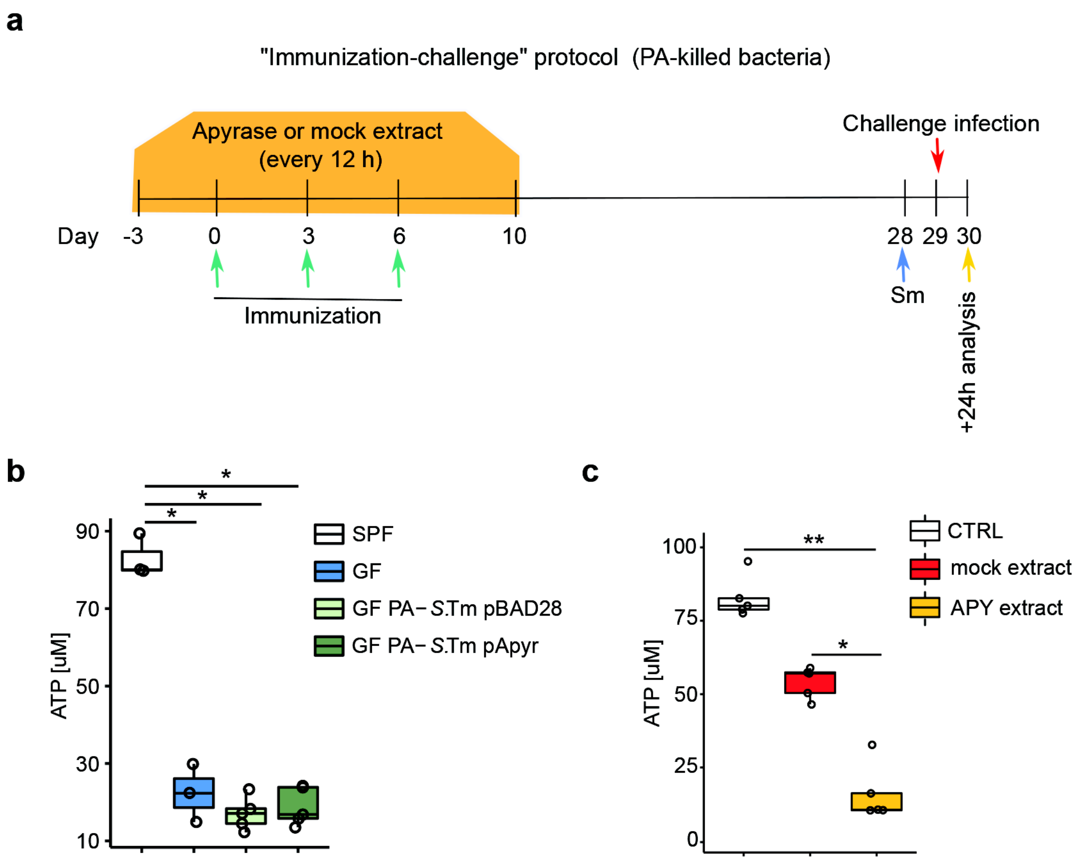

Supplementary Figure 8

Immunization-challenge protocol with PA-killed bacteria, lack of ATP release in the gut by PA-killed bacteria and *in vivo* apyrase activity of periplasmic extracts from *E. coli* transformants

(**a**) Diagram showing the immunization-challenge protocol with PA-killed bacteria. At day 0, 3 and 6 mice were gavaged with the different transformants. Where indicated, mice were conditioned with apyrase every 12 h as shown. On day 28, Sm was administered (25 mg i.g.); 24 h later (day 29) mice were infected with 10^8 CFU of *S*.Tm^{WT}. At 24 h post infection, mice were euthanized for analysis. (**b**) ATP quantification in ilea from untreated SPF or GF mice and GF mice at 12 h after orogastric administration of PA-*S*.Tm^{pBAD28} or PA- *S*.Tm^{pApyr}. (**c**) ATP quantification in ilea from SPF mice at 12 h after orogastric administration of PBS (CTRL), mock extract or apyrase extract. Kruskal–Wallis with Dunn's post-test. *p < 0.05,**p < 0.01. One representative experiment out of two is shown.

a

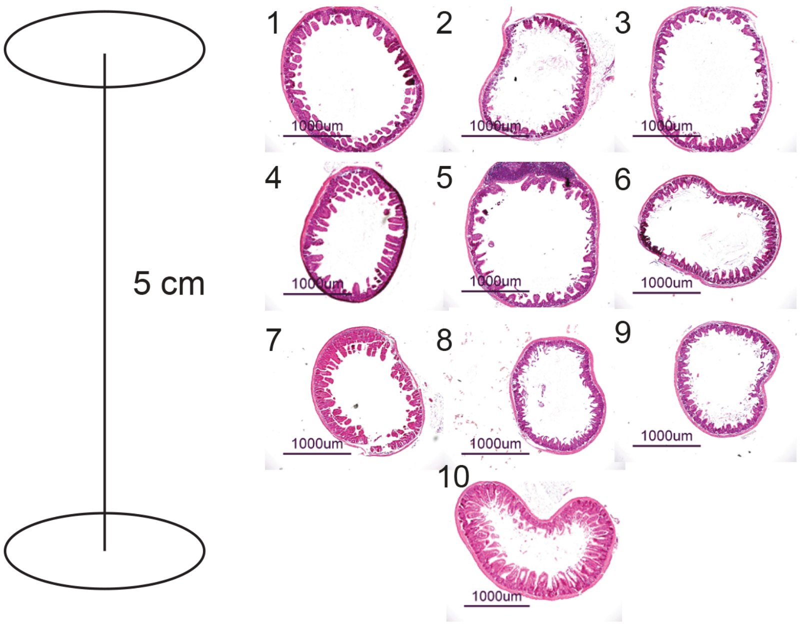

b

Area (mm²)		
**Sec.**	**Calc. (mm^2)**	**Corr. (mm^2)**
1	2,02	2,33
2	1,41	1,63
3	2,13	2,74
4	1,1	1,27
5	2,22	2,56
6	1,55	1,8
7	1,16	1,34
8	1,06	1,22
9	1,19	1,38
10	1,02	1,89

Ileal volume = 90 µl
Intestinal wash = 10 ml
Dil. Factor = 111
Actual ATP = Cal. ATP * Dil.Fact

Supplementary Figure 9
Ileal volume measurement
(**a**) Eight µm sections from terminal ileum used to calculate lumen area with ImageJ. (**b**) Obtained values either not corrected (Calc) or corrected (Corr) for shrinkage, as described in ref. 1 of this section.

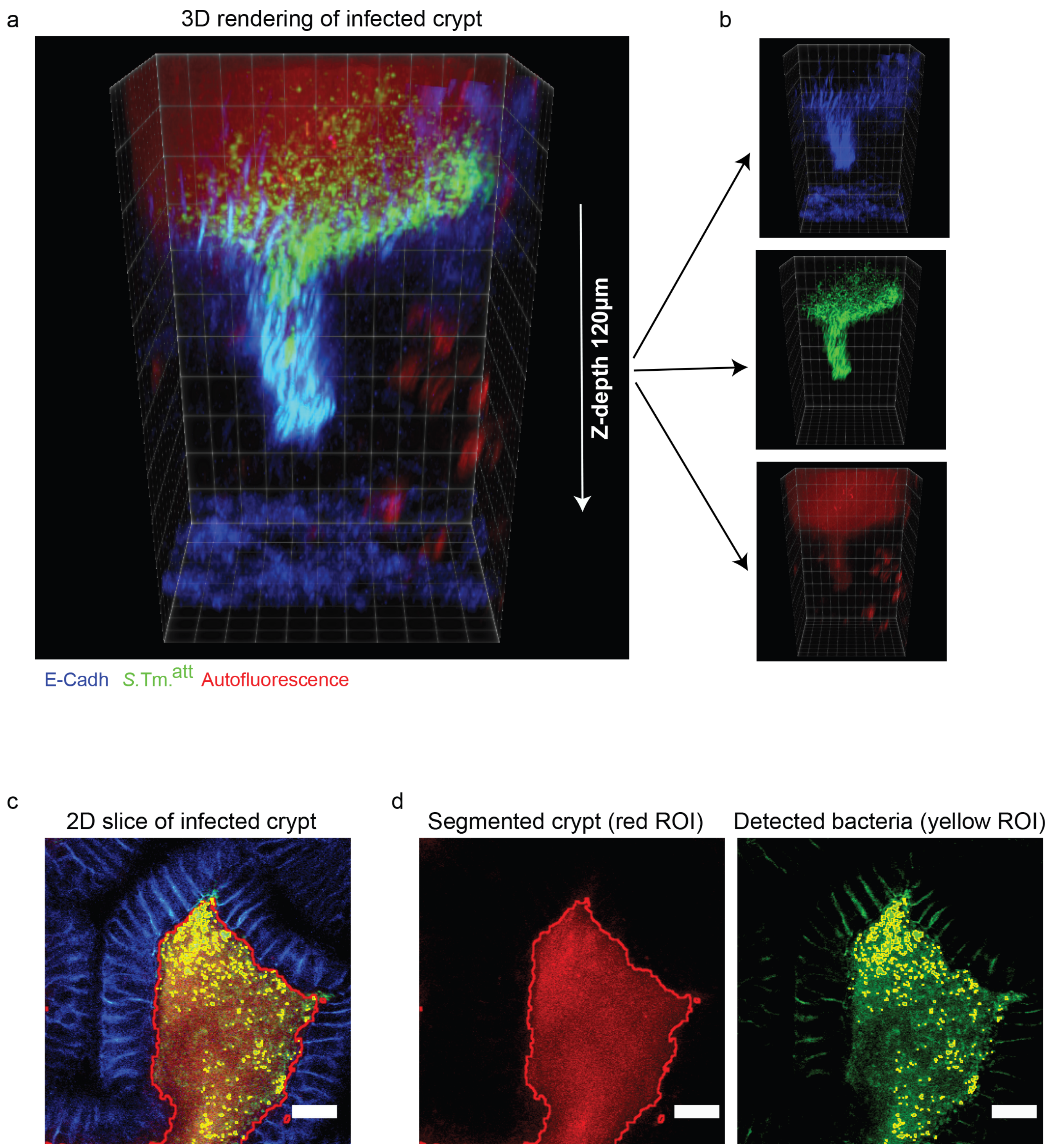

Supplementary Figure 10

Determination of infection extent by evaluating the total pathogen load in the caecal crypts using calculation of total fluorescence inside the caecal crypts area

(**a**) 3D rendering of 2-photon z-stack reconstruction of caecal crypt infected with *S*.Tm. E-Cadherin signal is shown in cyan, bacteria are shown in green, while mucus and macrophages auto-fluorescence is shown in red. The total depth of the reconstructed volume is 120 µm. (**b**) Three channel decomposition of the rendering shown in a. (**c**) Single slice of an infected crypt. The image shows merge of the three channel: E-Cadherin in cyan, mucus auto-fluorescence in the internal part of the crypt in red and bacteria in green. (**d**) Segmentation of the internal part of the crypt (red ROI) and detected bacteria (yellow ROI): only signal above threshold (see methods) and inside red ROI was measured. Scale bar, 20 µm.

Supplementary Table 1. Bacterial strains used in this study

Strain	Genotype information	Resistance	Reference
E.Coli[pBAD28]	*E. coli* DH10b transformed with the arabinose-inducible PBAD expression vector	Cm, Amp	2
E.Coli[pApyr]	*E. coli* DH10b transformed with pBAD28 carrying *phoN2*::HA	Cm, Amp	3
*S.*Tm[WT]	SB300 *S. enterica* serovar Typhimurium SL1344 (wildtype)	Sm	4
*S.*Tm[pBAD28]	Avirulent *gyrA1816 Δcya1 Δcrp1 S.*Tm (ATCC53648) transformed with the arabinose-inducible PBAD expression vector	Cm, Amp	This study
*S.*Tm[pApyr]	Avirulent *gyrA1816 Δcya1 Δcrp1 S.*Tm (ATCC53648) transformed with pBAD28 carrying *phoN2::*HA	Cm, Amp	This study
*S.*Tm[att] mCherry	SB300 derivate, *ΔinvG ΔsseD S.*Tm M2702 transformed with P*rpsM*::mCherry (pFPV25.1)	Amp	5
*S.*Tm[att] GFP	SB300 derivate, *ΔinvG ΔsseD S.*Tm M2702 transformed with P*rpsM*::*gfp*mut2 (pM965)	Amp	6

Cm: Chloramphenicol 30µg/ml; Amp: Ampicillin 100µg/ml; Sm: Streptomycin 50µg/ml.

References

1. Casteleyn C, Rekecki A, Van der Aa A, Simoens P, Van den Broeck W. Surface area assessment of the murine intestinal tract as a prerequisite for oral dose translation from mouse to man. *Lab Anim* 2010, **44**(3)**:** 176-183.
2. Guzman LM, Belin D, Carson MJ, Beckwith J. Tight regulation, modulation, and high-level expression by vectors containing the arabinose PBAD promoter. *Journal of bacteriology* 1995, **177**(14)**:** 4121-4130.
3. Santapaola D, Del Chierico F, Petrucca A, Uzzau S, Casalino M, Colonna B*, et al.* Apyrase, the product of the virulence plasmid-encoded *phoN2* (*apy*) gene of *Shigella flexneri*, is necessary for proper unipolar IcsA localization and for efficient intercellular spread. *Journal of bacteriology* 2006, **188**(4)**:** 1620-1627.
4. Hoiseth SK, Stocker BA. Aromatic-dependent *Salmonella* typhimurium are non-virulent and effective as live vaccines. *Nature* 1981, **291**(5812)**:** 238-239.
5. Drecktrah D, Levine-Wilkinson S, Dam T, Winfree S, Knodler LA, Schroer TA*, et al.* Dynamic behavior of *Salmonella*-induced membrane tubules in epithelial cells. *Traffic* 2008, **9**(12)**:** 2117-2129.
6. Stecher B, Hapfelmeier S, Muller C, Kremer M, Stallmach T, Hardt WD. Flagella and chemotaxis are required for efficient induction of *Salmonella enterica* serovar Typhimurium colitis in streptomycin-pretreated mice. *Infection and immunity* 2004, **72**(7)**:** 4138-4150.
