# Supplementary material for: ATP released by intestinal bacteria limits the generation of protective IgA against enteropathogens
Source: Nat Commun. 2019 Jan 16;10:250. doi: 10.1038/s41467-018-08156-z (PMC6335424; doi:10.1038/s41467-018-08156-z)
Supplement: Supplementary file 2 — Reporting Summary [file 41467_2018_8156_MOESM2_ESM.pdf]

natureresearch

Corresponding author(s): Fabio Grassi

# Reporting Summary

Nature Research wishes to improve the reproducibility of the work that we publish. This form provides structure for consistency and transparency in reporting. For further information on Nature Research policies, see Authors & Referees and the Editorial Policy Checklist.

## Statistical parameters

When statistical analyses are reported, confirm that the following items are present in the relevant location (e.g. figure legend, table legend, main text, or Methods section).

| n/a | Confirmed | |
|---|---|---|
| ☐ | ☒ | The exact sample size (*n*) for each experimental group/condition, given as a discrete number and unit of measurement |
| ☐ | ☒ | An indication of whether measurements were taken from distinct samples or whether the same sample was measured repeatedly |
| ☐ | ☒ | The statistical test(s) used AND whether they are one- or two-sided<br>*Only common tests should be described solely by name; describe more complex techniques in the Methods section.* |
| ☒ | ☐ | A description of all covariates tested |
| ☐ | ☒ | A description of any assumptions or corrections, such as tests of normality and adjustment for multiple comparisons |
| ☐ | ☒ | A full description of the statistics including central tendency (e.g. means) or other basic estimates (e.g. regression coefficient) AND variation (e.g. standard deviation) or associated estimates of uncertainty (e.g. confidence intervals) |
| ☒ | ☐ | For null hypothesis testing, the test statistic (e.g. *F*, *t*, *r*) with confidence intervals, effect sizes, degrees of freedom and *P* value noted<br>*Give P values as exact values whenever suitable.* |
| ☒ | ☐ | For Bayesian analysis, information on the choice of priors and Markov chain Monte Carlo settings |
| ☒ | ☐ | For hierarchical and complex designs, identification of the appropriate level for tests and full reporting of outcomes |
| ☒ | ☐ | Estimates of effect sizes (e.g. Cohen's *d*, Pearson's *r*), indicating how they were calculated |
| ☐ | ☒ | Clearly defined error bars<br>*State explicitly what error bars represent (e.g. SD, SE, CI)* |

*Our web collection on statistics for biologists may be useful.*

## Software and code

Policy information about availability of computer code

| | |
|---|---|
| Data collection | No software was used |
| Data analysis | FlowJo software (TreeStar, Ashland, OR), FACS Diva software (BD Biosciences), FIJI software, GraphPad Prism 7.02 |

For manuscripts utilizing custom algorithms or software that are central to the research but not yet described in published literature, software must be made available to editors/reviewers upon request. We strongly encourage code deposition in a community repository (e.g. GitHub). See the Nature Research guidelines for submitting code & software for further information.

## Data

Policy information about availability of data

All manuscripts must include a data availability statement. This statement should provide the following information, where applicable:

- Accession codes, unique identifiers, or web links for publicly available datasets
- A list of figures that have associated raw data
- A description of any restrictions on data availability

The authors declare that the main data supporting the findings of this study are available within the article and its Supplementary Information files. Extra data are available at 10.5072/zenodo.254203 [https://sandbox.zenodo.org/record/254203#.XAVeYCdRe3I].

# Field-specific reporting

Please select the best fit for your research. If you are not sure, read the appropriate sections before making your selection.

☒ Life sciences ☐ Behavioural & social sciences ☐ Ecological, evolutionary & environmental sciences

For a reference copy of the document with all sections, see nature.com/authors/policies/ReportingSummary-flat.pdf

# Life sciences study design

All studies must disclose on these points even when the disclosure is negative.

| | |
|---|---|
| Sample size | Sample size was determined using the resource equation method approach |
| Data exclusions | No data were excluded from the analysis |
| Replication | All attempts of replication of experimental results were successful |
| Randomization | Animals were randomly allocated for immunization and infection |
| Blinding | Histopathological analysis was performed in a blinded manner |

# Reporting for specific materials, systems and methods

## Materials & experimental systems

| n/a | Involved in the study | |
|---|---|---|
| ☐ | ☒ | Unique biological materials |
| ☐ | ☒ | Antibodies |
| ☒ | ☐ | Eukaryotic cell lines |
| ☒ | ☐ | Palaeontology |
| ☐ | ☒ | Animals and other organisms |
| ☒ | ☐ | Human research participants |

## Methods

| n/a | Involved in the study | |
|---|---|---|
| ☒ | ☐ | ChIP-seq |
| ☐ | ☒ | Flow cytometry |
| ☒ | ☐ | MRI-based neuroimaging |

## Unique biological materials

Policy information about availability of materials

| | |
|---|---|
| Obtaining unique materials | All materials are available for use by others upon request and Institute for Research in Biomedicine sponsored projects approval. |

## Antibodies

| | |
|---|---|
| Antibodies used | Details of all antibodies used are listed in the Methods section. |
| Validation | Specificities of antibodies are validated by data provided in the manuscript. |

## Animals and other organisms

Policy information about studies involving animals; ARRIVE guidelines recommended for reporting animal research

| | |
|---|---|
| Laboratory animals | We used laboratory mouse (Mus musculus) in this study; they were all in a C57BL/6 background, female and male, 8 weeks of age. |
| Wild animals | n/a |
| Field-collected samples | n/a |

## Flow Cytometry

### Plots

Confirm that:

☒ The axis labels state the marker and fluorochrome used (e.g. CD4-FITC).

☒ The axis scales are clearly visible. Include numbers along axes only for bottom left plot of group (a 'group' is an analysis of identical markers).

☒ All plots are contour plots with outliers or pseudocolor plots.

☒ A numerical value for number of cells or percentage (with statistics) is provided.

### Methodology

| | |
|---|---|
| Sample preparation | A description of the sample preparations for flow cytometry is detailed in the Methods section. |
| Instrument | We used a LSR Fortessa flow cytometry machine (BD Biosciences). |
| Software | We used the software FACSDiva (BD Biosciences) to collect the data and the software FlowJo v10 (Treestar Inc.) to analyze the data. |
| Cell population abundance | n/a |
| Gating strategy | For lymphocytes, we first selected lymphocytes based on FSCxSSC; then, we selected the singlets based on SSC-H x SSC-W. After, we selected Tfh cells based on CD4xCXCR5xICOS gating; we selected GC B cells based on CD19xB220xFasxPNA gating; we selected LPS specific plasma cells based on CD138xIgA gating. For bacteria, we first selected bacteria based on FSC and SSC in logarithmic mode; bacteria integrity was evaluated based on DAPIxDIBAC and DAPIxSybrGreen gating and IgA coating based on IgAxSybrGreen gating. |

☒ Tick this box to confirm that a figure exemplifying the gating strategy is provided in the Supplementary Information.
